# Effect of nanoparticles on heat capacity of nanofluids based on molten salts as PCM for thermal energy storage

**DOI:** 10.1186/1556-276X-8-448

**Published:** 2013-10-29

**Authors:** Manila Chieruzzi, Gian F Cerritelli, Adio Miliozzi, José M Kenny

**Affiliations:** 1 Civil and Environmental Engineering Department, UdR INSTM, University of Perugia, Strada di Pentima, 4, 05100 Terni, Italy; 2 ENEA – Italian National Agency for New Technologies, Energy and Sustainable Economic Development, Casaccia Research Centre, Via Anguillarese, 301 - 00123 S. Maria di Galeria, Rome, Italy; 3 Institute for Polymer Science and Technology, ICTP-CSIC, Juan De la Cierva, 3, 28006 Madrid, Spain

**Keywords:** Phase change materials, Nanofluid, Thermal energy storage, Nanoparticles, Heat capacity, Molten salt, Nanocomposite

## Abstract

In this study, different nanofluids with phase change behavior were developed by mixing a molten salt base fluid (selected as phase change material) with nanoparticles using the direct-synthesis method. The thermal properties of the nanofluids obtained were investigated. These nanofluids can be used in concentrating solar plants with a reduction of storage material if an improvement in the specific heat is achieved. The base salt mixture was a NaNO_3_-KNO_3_ (60:40 ratio) binary salt. The nanoparticles used were silica (SiO_2_), alumina (Al_2_O_3_), titania (TiO_2_), and a mix of silica-alumina (SiO_2_-Al_2_O_3_). Three weight fractions were evaluated: 0.5, 1.0, and 1.5 wt.%. Each nanofluid was prepared in water solution, sonicated, and evaporated. Measurements on thermophysical properties were performed by differential scanning calorimetry analysis and the dispersion of the nanoparticles was analyzed by scanning electron microscopy (SEM). The results obtained show that the addition of 1.0 wt.% of nanoparticles to the base salt increases the specific heat of 15% to 57% in the solid phase and of 1% to 22% in the liquid phase. In particular, this research shows that the addition of silica-alumina nanoparticles has a significant potential for enhancing the thermal storage characteristics of the NaNO_3_-KNO_3_ binary salt. These results deviated from the predictions of the theoretical model used. SEM suggests a greater interaction between these nanoparticles and the salt.

## Background

The growing world energy demand [1] is increasing the burning of fossil fuels and, consequently, the carbon dioxide emissions. In order to limit these emissions, it is necessary to make better use of the produced thermal energy by increasing the energy efficiency of industrial processes (heat recovery) and buildings and the use of renewable sources such as solar energy [2].

Economic storage of thermal energy (thermal energy storage - TES) is a key technological issue for solar thermal power plants and industrial waste heat recovery [3-5]. The overall objectives of heat storage integration are to increase the solar contribution, to improve efficiency, and to reduce the levelized energy cost (LEC). TES systems can be used at high temperature (T > 400°C) or at lower temperature (ranging from 100°C to about 300°C) for heat and solar cooling. Low-temperature storage systems are based almost entirely on sensible heat storage using liquid water [3], but for temperatures exceeding 100°C (concentrated solar power plants), non-pressurized liquid water and the use of pressure vessels make this technology unattractive.

Among the various methods of energy storage, latent heat thermal energy storage (LHTES) systems using phase change materials (PCMs) have been gaining importance in such fields as solar energy systems, district heating and cooling systems, energy efficiency buildings [6-9], cool storage systems for central air-conditioning systems, and waste heat recovery systems [10]. This is mainly due to their high energy storage density and their ability to provide heat at a constant temperature. Since the latent heat of fusion between the liquid and solid phases of PCMs is high compared to sensible heat, storage systems utilizing PCMs can be reduced in size with respect to systems based on sensible heat. Therefore, several studies on PCM used as thermal energy storage material have been published [10-16].

The materials needed for phase-change thermal energy storage must have a large latent heat and high thermal conductivity. They should have a melting temperature lying in the practical range of operation, offer chemical stability and they should be low cost, nontoxic, and non-corrosive. PCMs studied during the last 40 years are mainly paraffin waxes, fatty acids, metal alloys, salts (fluorides and chlorides, hydroxides, nitrates, carbonates) [17-20]. There are many LHTES systems used in practical applications. They can be composed of PCM elements with similar shapes such as PCM spheres, PCM cylinders, PCM flat plates or even PCM capsules with irregular shapes [11].

Some researchers have shown that the addition of nanoparticles may allow an increase in both the thermal capacity and the thermal conductivity of the storage media [21-23]. The concept of nanofluid as a fluid with nanoparticles suspended by Brownian motion was introduced in 1995 by Choi who showed an increase in thermal properties of some fluids after the addition of copper or aluminum nanoparticles [24]. The increase of these properties can be considerably enhanced by a small amount of nanoparticles added to the storage media with obvious advantages for the thermal energy storage systems. In fact, a high thermal capacity allows storing a high quantity of heat in a small volume of material. This allows the realization of compact thermal storage systems with the advantage to limit energy losses (reducing the outer exchanging surfaces) and, above all, to reduce costs.

However, research work on the specific heat of nanofluids has been limited compared to that on thermal conductivity especially those prepared by adding nanoparticles [21,25-29]. Nevertheless, it is essential to evaluate the heat capacity of these nanofluids since an improvement in the specific heat can reduce the amount of storage material.

The aim of this work is the development of a new phase change material using different kinds of nanoparticles embedded in a molten salt base material and the thermal characterization of the nanofluid obtained.

## Methods

### Materials

A binary nitrate salt, widely used in liquid phase as sensible heat storage media in high temperature solar plants (solar salt), has been selected as PCM. This salt is a mixture of 60% NaNO_3_ and 40% KNO_3_ which has a melting point of about 220°C. This melting temperature is useful for many medium-low power applications [5]. The salts were purchased from Sigma-Aldrich (St. Louis, MO, USA). The selected nanoparticles were silica (Aerosil300, average diameter 7 nm, Evonik Industries, Hanau-Wolfgang Germany), alumina (Aeroxide AluC, average diameter 13 nm), a hydrophilic fumed mixed oxide of silica (82% to 86%) and alumina (14% to 18%) (Aerosil Cok84, average diameter 2 to 200 nm) supplied by Evonik and titania (titanium dioxide, 99.97% average diameter 20 nm) supplied by Sigma-Aldrich.

### Experimental procedures

The binary salt was prepared by mixing 60 parts of NaNO_3_ with 40 parts of KNO_3_ in solid state, then heating them at 300°C to achieve a complete melting and mixing of the two salts. The system was then cooled at room temperature and a white solid mixture was obtained. The solid binary salt was then milled to powder and immediately used. The nanoparticles were dispersed into three concentrations: 0.5, 1.0, and 1.5 wt.%. The procedure followed to prepare the nanofluids consists of four steps: 198 mg of NaNO_3_-KNO_3_ and 2 mg of nanoparticles (related in this case to 1.0 wt.% of nanoparticles) were measured on an analytical balance with ±0.1-mg precision (Mettler Toledo, type AB104-S, Greifensee, Switzerland); the binary salt and the nanoparticles were dispersed in 20 ml of distilled water by ultrasonic mixing for 100 min (Ultrasonic bath, EMMEGI mod.AC-5, Milan, Italy); afterwards, the water solution was heated at 200°C on a hot plate to fully remove the water (for at least 2 h) [21]. The same procedure was followed to prepare the nanofluids with 0.5 and 1.5 wt.% of nanoparticles and to prepare the neat molten salt for comparison with the nanofluids obtained. The nanofluids were formulated without the use of any dispersant/surfactant. All the materials used in this work were subjected to dehydration in order to remove all the water entrapped. After this operation, they were immediately used for DSC analysis. In this way, we avoided any possible negative effect of the water.

### Differential scanning calorimetry

Differential scanning calorimetry (DSC) tests were performed on a Mettler-Toledo DSC 822E/400. The dry samples obtained after water evaporation were introduced in standard aluminum pans with lid and subjected to the following thermal cycle in nitrogen atmosphere: held at 150°C for 5 min (to remove any absorbed water in the sample), heating from 150°C to 300°C at 20°C/min, held at 300°C for 5 min, cooling from 300°C to 150°C at 20°C/min. Six consecutive cycles were run on each sample without opening the DSC furnace to ensure good mixing of the sample and reproducibility of the results. In this way, the effect of the initial granulometry was also lost after the first cycles. The DSC thermograms were analyzed and the phase-change heat and melting temperatures were obtained using the software STARe. Moreover, the calorimetric data were used to calculate the specific heat (C_p_) of the samples.

Same scanning tests were performed on the base salt and the melting properties compared with those obtained with the nanofluids. In this way, the effect of the different nanoparticles could be evaluated.

In particular, the DSC procedure followed to estimate specific heat values was the three-step procedure [30]. First of all, a thermal cycle was fixed. In the first step, a measurement was taken with two empty sample pans. During this measurement, the baseline heat flux (Q_0_) was obtained. The results of this measurement indicate the bias in the DSC. In the second step, a pan containing the reference sample with a known specific heat and an empty pan were loaded into the calorimeter. The heat flux recorded was *Q*_ref_. In the third step, a pan containing the sample and an empty pan were loaded into the calorimeter. The heat flux into the sample (*Q*_sample_) was recorded. The specific heat of the sample was obtained as follows: C_p,sample_ = [(*Q*_sample_−*Q*_0_)/(*Q*_ref_−*Q*_0_)] × (*m*_ref_−*m*_sample_) × C_p,ref_, where *m*_ref_ and *m*_sample_ indicate the masses of the reference and sample, respectively.

This procedure was validated with our base fluid whose specific heat is well known.

The measurements were also compared with a theoretical model based on the assumption of thermal equilibrium between the particles and the surrounding fluid [26,31]:

(1) $$C_{p,\mathrm{nf}}=\frac{\rho_{\mathrm{np}}\phi_{\mathrm{np}}C_{p,\mathrm{np}}+\rho_{f}\phi_{f}C_{p,f}}{\rho_{\mathrm{np}}\phi_{\mathrm{np}}+\rho_{f}\phi_{f}},$$

where C_p_ is specific heat, *∅* is the volume fraction, *ρ* is the density, and the subscripts np, nf, and f refer to nanoparticle, nanofluid, and base fluid, respectively.

### Scanning electron microscopy

The morphological analysis of the samples was performed with a field emission scanning electron microscope (FESEM model SUPRA25, ZEISS, Oberkochen, Germany). One specimen for each system after DSC measurement was analyzed in order to evaluate the dispersion of the nanoparticles. Each sample was metallized with a thin layer of gold (15 nm, 99.99% of gold, 2 × 10^−6^ Torr) in a thermal evaporator (Sistec thin film equipment model GP 20 by Kenosistec Angelantoni Group, Massa Martana (PG), Italy). For the surface analysis, secondary electrons were used. SEM analysis has been used in other works focused in the preparation and characterization of nanofluids to evaluate the dispersion/aggregation of nanoparticles [21,22,32].

Moreover, a recent study analyzed the aggregation of nanoparticles in the same base fluid studied in our work using the SEM technique [33].

## Results and discussion

### Calorimetric analysis

**Figure 1:**
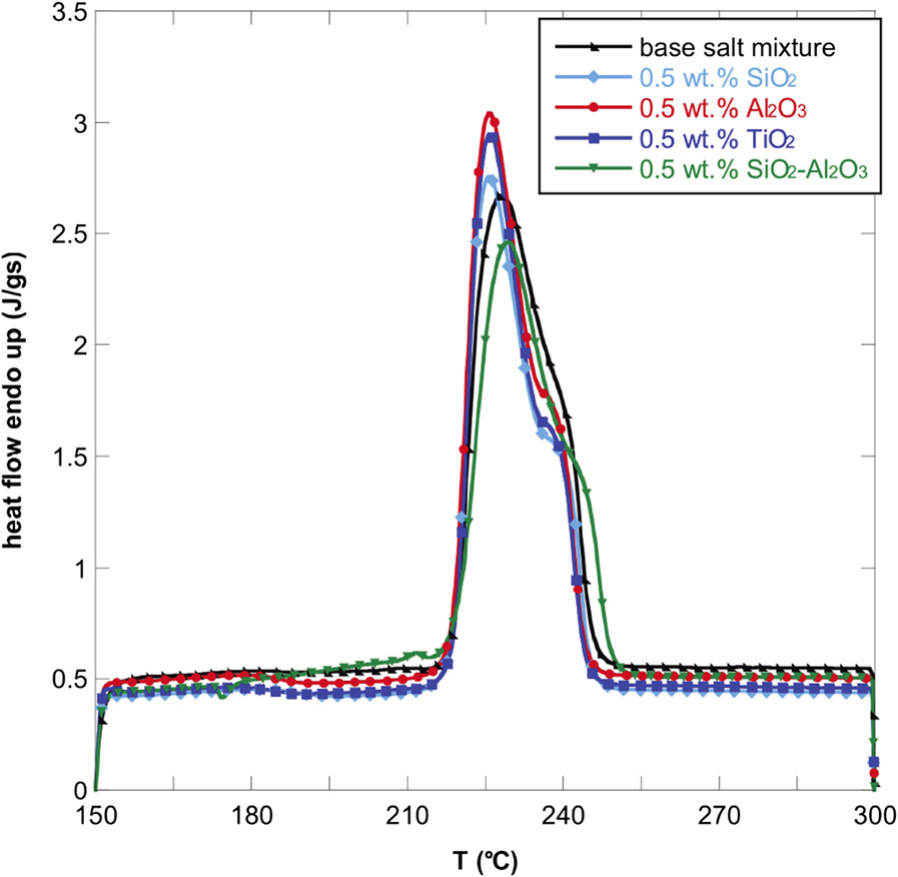
**Heat flow versus temperature for NaNO**_**3**_-**KNO**_**3 **_**binary salt mixture and nanofluids** (**0**.**5 wt.% of oxide nanoparticles).**

**Figure 2:**
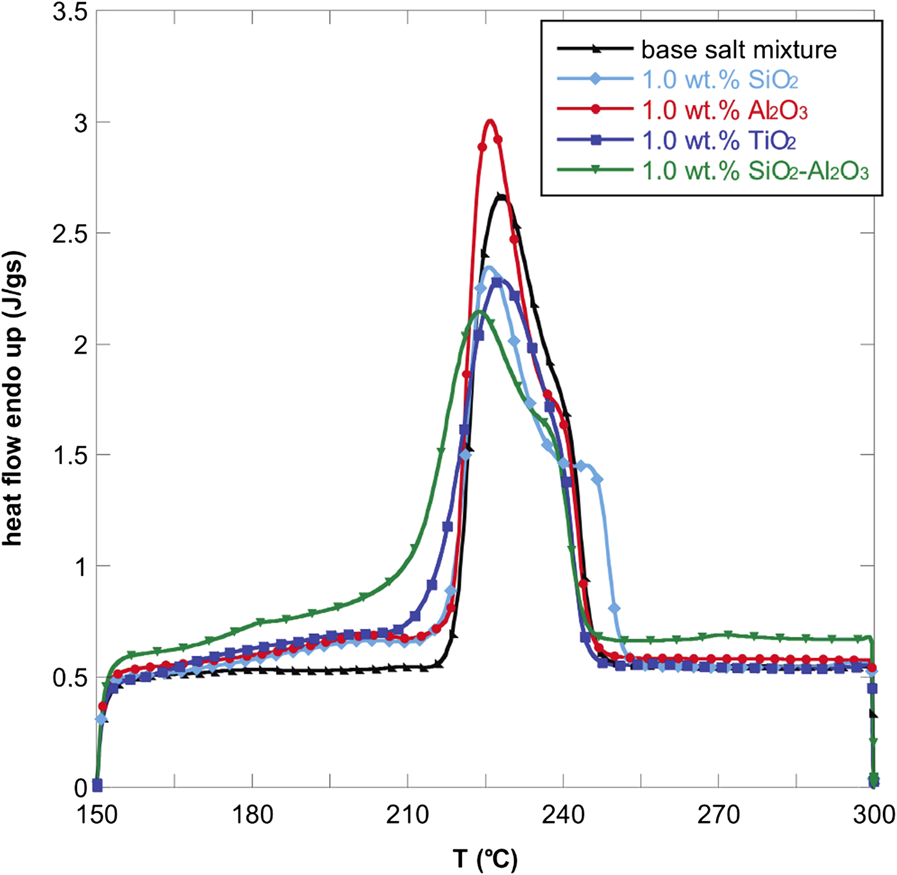
**Heat flow versus temperature for NaNO**_**3**_**-KNO**_**3 **_**binary salt mixture and nanofluids (1.0 wt.% of oxide nanoparticles).**

**Figure 3:**
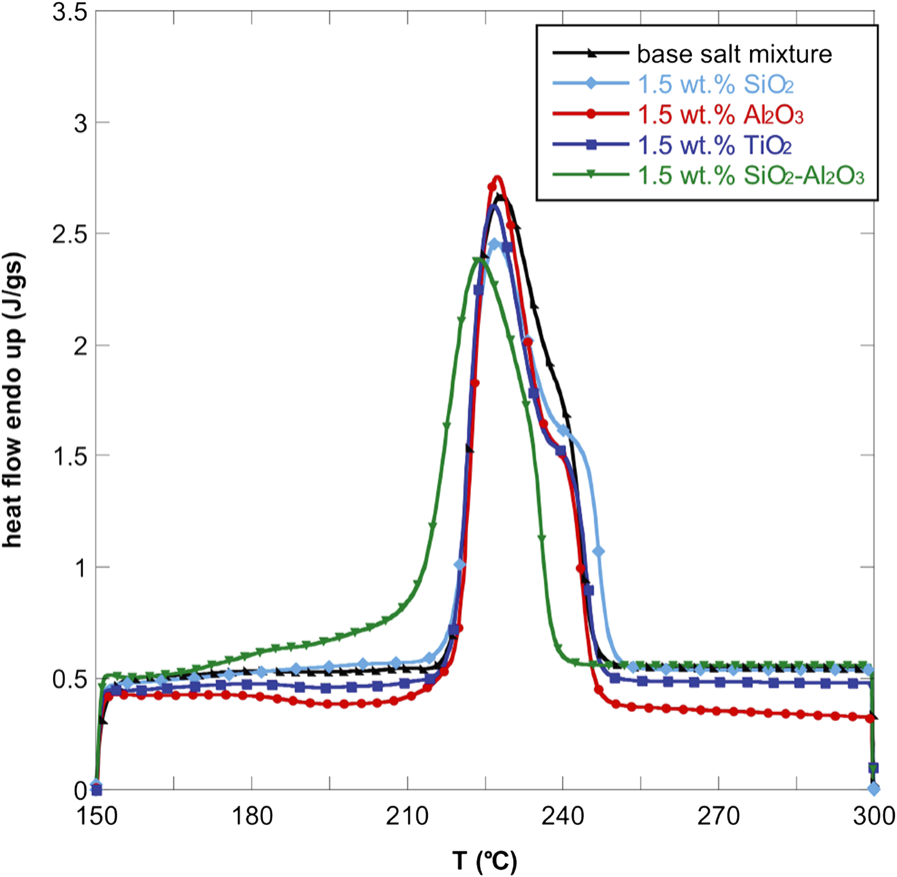
**Heat flow versus temperature for NaNO**_**3**_-**KNO**_**3 **_**binary salt mixture and nanofluids (1.5 wt.% of oxide nanoparticles).**

Figures 1, 2, and 3 show the DSC thermograms of the NaNO_3_-KNO_3_ salt mixture and the nanofluids with different nanoparticles and concentrations. As it can be seen, the different nanoparticles induce a change in the shape of the heat flow curve of the base salt. To evaluate the effect of the nanoparticles on the phase-change curve, the heat of fusion, the melting point, and the onset temperatures of all samples were obtained from DSC measurements and reported in Table 1. The onset temperature was taken as the intersection point of the baseline before transition and the inflectional tangent while the melting points were calculated taking into account the fact that the peaks are not symmetric. In particular, the area under the curve was divided into two equal parts and the corresponding temperature taken as melting point. The temperatures were reported in Table 1.

**Table 1:** **Heat of fusion**, **melting temperature**, **and onset temperature of NaNO**_**3**_-**KNO**_**3**_ (**60**:**40**) **mixture and nanofluids obtained with different nanoparticles**

**Material**	**Nanoparticle concentration (wt.%)**	**Heat of fusion (kJ/kg)**	**Onset temperature (°C)**	**Melting temperature (°C)**
Base salt mixture	-	110.01	219.88	232.01
Base salt + SiO_2_	0.5	109.20	219.48	230.20
	1.0	126.39	218.84	230.44
	1.5	114.40	219.47	231.80
Base salt + Al_2_O_3_	0.5	115.45	219.25	229.95
	1.0	127.11	219.25	229.33
	1.5	116.83	219.84	230.51
Base salt + TiO_2_	0.5	110.14	219.78	229.92
	1.0	115.26	219.89	230.80
	1.5	105.36	219.65	230.78
Base salt + SiO_2_-Al_2_O_3_	0.5	112.82	219.70	232.23
	1.0	127.24	209.32	223.89
	1.5	114.06	211.51	224.01

In particular, the analyzed binary salt and the nanofluids obtained by adding nanoparticles in the binary salt, are not pure materials; therefore, instead of a melting temperature, they show a range of melting temperatures. The onset and the melting temperatures were both taken because the complete phase transformation is not reached at the onset temperature but only above the melting temperature. In fact, above this temperature, the salt mixture and the nanofluids are completely liquid and can be used as fluids for solar energy storage. For this reason, the melting temperature was taken as the real melting point of the salt mixture and the nanofluids and the two characteristic temperatures were both useful to describe the melting behavior of PCMs.

The onset temperature of the endothermic peak for the binary salt was 219.8°C while the melting temperature was 232.01°C. The addition of the four types of nanoparticles lead to a reduction of onset temperatures and melting points. This effect was found for all the three concentrations investigated but it was more evident for 1.0 wt.% of nanoparticles added. In particular, the addition of 1.0 wt.% of SiO_2_-Al_2_O_3_ lead to a maximum Tonset decrease of 5% with respect to the base salt mixture (i.e., about 10°C).

In particular, the onset temperature decreased by adding SiO_2_, Al_2_O_3_, TiO_2_, and SiO_2_-Al_2_O_3_. This means that the phase change occurs at a lower temperature with respect to the base salt with a clear advantage when used in solar plants.

Moreover, also the phase-change temperature was lowered by adding the selected nanoparticles to the NaNO_3_-KNO_3_ salt mixture, especially with 1.0 wt.% of SiO_2_-Al_2_O_3_. In this case, the melting point decreased by more than 8°C. For the other nanofluids, the decrease in melting temperature was slightly modified (<1%).

A higher effect of the addition of nanoparticles was obtained on the latent heat. In particular, adding 1.0 wt.% of the nanoparticles produced an enhancement of the heat of fusion by more than 15% for almost all the nanofluids obtained. Only the nanofluid based on TiO_2_ produced an increment of 5%. Even in this case, 1.0 wt.% of SiO_2_-Al_2_O_3_ produced the higher enhancement. This means that the nanoparticles added to the base salt mixture allow the heat storage to be more effective per unit volume.

The addition of nanoparticles influenced also the heat capacity in solid and liquid phases. The specific heats were calculated as average of single values recorded in the temperature range 155°C to 220°C (where 220°C is about the onset temperature) in solid phase and in the range 250°C to 300°C (where 250°C is about the endset temperature) in liquid phase (the endset temperature is the intersection point of the baseline after transition and the inflectional tangent).

Table 2 shows the C_p_ values that represent the average of at least three measurements. The average specific heat of the base salt in the liquid phase is 1.648 kJ/kg·K. The addition of nanoparticles to base salt increased the C_p_ both in solid and liquid phase with 1.0 wt.% of all nanoparticles analyzed. The higher increase of C_p_ was obtained in solid phase; in this case, there is a slight increase of C_p_ with temperature) with respect to the liquid phase (where the C_p_ is almost constant with temperature) [17,33].

**Table 2:** **Specific heat of NaNO**_**3**_-**KNO**_**3**_ (**60**:**40**) **mixture and nanofluids obtained with different nanoparticles**

**Material**	**Nanoparticle concentration wt.%**	**Specific heat (solid phase; kJ**/**kg**·**K**)	**Enhancement (solid phase; %)**	**Specific heat (liquid phase; kJ/kg·K)**	**Enhancement (liquid phase; %)**
Base salt mixture	-	1.604	-	1.648	-
Base salt + SiO_2_	0.5	1.341	−16.4	1.329	−19.3
	1.0	1.843	14.9	1.661	0.8
	1.5	1.635	2.0	1.624	−1.4
Base salt + Al_2_O_3_	0.5	1.526	−4.8	1.522	−7.6
	1.0	1.923	19.9	1.745	5.9
	1.5	1.550	−3.3	1.590	−3.5
Base salt + TiO_2_	0.5	1.372	−14.4	1.390	−15.6
	1.0	1.508	−6.0	1.544	−6.3
	1.5	1.432	−10.7	1.454	−11.8
Base salt + SiO_2_-Al_2_O_3_	0.5	1.572	−2.0	1.525	−7.5
	1.0	2.529	57.7	2.018	22.5
	1.5	2.162	34.8	1.673	1.5

Furthermore, the C_p_ increase obtained with the addition of 1.0 wt.% of SiO_2_-Al_2_O_3_ nanoparticles was undoubtedly the most important among all the characterized mixtures (57% in solid phase and 22% in liquid phase).

The best enhancement of thermal properties was obtained with the addition of 1.0 wt.% of nanoparticles as expected [25,26,33,34].

**Figure 4:**
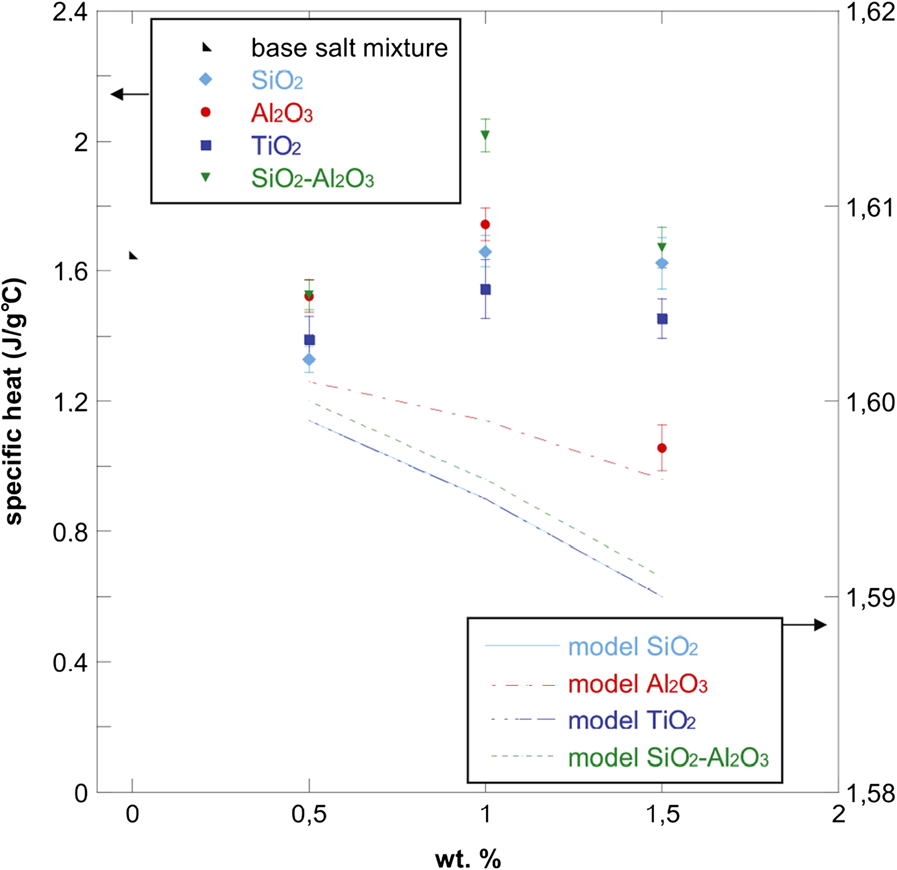
**Specific heat values versus particle concentration for NaNO**_**3**_-**KNO**_**3 **_**binary salt mixture and nanofluids.** Experimental data and model predictions.

Figure 4 shows the theoretical prediction of specific heats using Equation 1 for the nanofluids with SiO_2_, Al_2_O_3_, TiO_2_, and SiO_2_-Al_2_O_3_ nanoparticles in the three concentrations considered (0.5, 1.0, and 1.5 wt.%). Experimental values of C_p_ (reported in Table 2) were also plotted for all nanofluids and base salt mixture with the error bars. The nanoparticle density and C_p_ values used in this model were 2650, 3970, 4230, and 2860 Kg/m^3^ and 0.70, 1.10, 0.69, and 0.80 kJ/kg·K for SiO_2_, Al_2_O_3_, TiO_2_, and SiO_2_-Al_2_O_3_, respectively. For the base salt, a density value of 1,794 kg/m^3^ was considered while the C_p_ is the one reported in Table 2. As it can be seen, the values obtained with Equation 1 for all nanofluids are lower than the specific heat of the base salt mixture.

The reduction in the specific heat capacity of the nanofluids obtained from the model is clearly explained since oxide nanoparticles show lower specific heats with respect to the base phase change material. This behavior was also reported in previous studies [21,26,35,36].

Moreover, the values of C_p_ experimentally evaluated for 1.0 wt.% of nanoparticles resulted much higher than the theoretical values. This different behavior was explained by admitting the existence of other mechanisms for nanofluids [21]. Different mechanisms were proposed to explain the enhancement of thermal conductivity; it is possible to suppose that the same mechanisms could be used to explain the enhancement of specific heat. They are based on Brownian motion of nanoparticles [37-39], aggregation of nanoparticles [40-42] and formation of a nanolayer [21,43-45].

In our work, the observed enhancement of heat capacity was not related to the presence of a substructure. In any case, since it was shown that the specific heat is a structure-insensitive property [46], the formation of networks in the structure of nanofluids should not affect the specific heat.

In addition, the Brownian motion of nanoparticles should not be responsible for the enhancement since heat capacity is not a transport property. On the other hand, the agglomeration of nanoparticles and the formation of clusters can increase the thermal conductivity but, in our work, does not seem to have the same effect on the specific heat.

The most significative mechanism that led to an increment of C_p_ could be considered the formation of a solid-like nanolayer on the surface of the nanoparticle. This layer has higher thermal properties than the bulk liquid contributing in this way to the increase of specific heat of nanofluids [21,33].

The kinetic effect was taken into account in calorimetric analysis. The scanning rate adopted was 20°C/min because it was the heating rate commonly used for specific heat evaluation. Moreover, faster program rates seem to improve also the accuracy of specific heat evaluation [47]. DSC tests at 10°C/min were also performed and a difference of about 6% in C_p_ was found.

**Figure 5:**
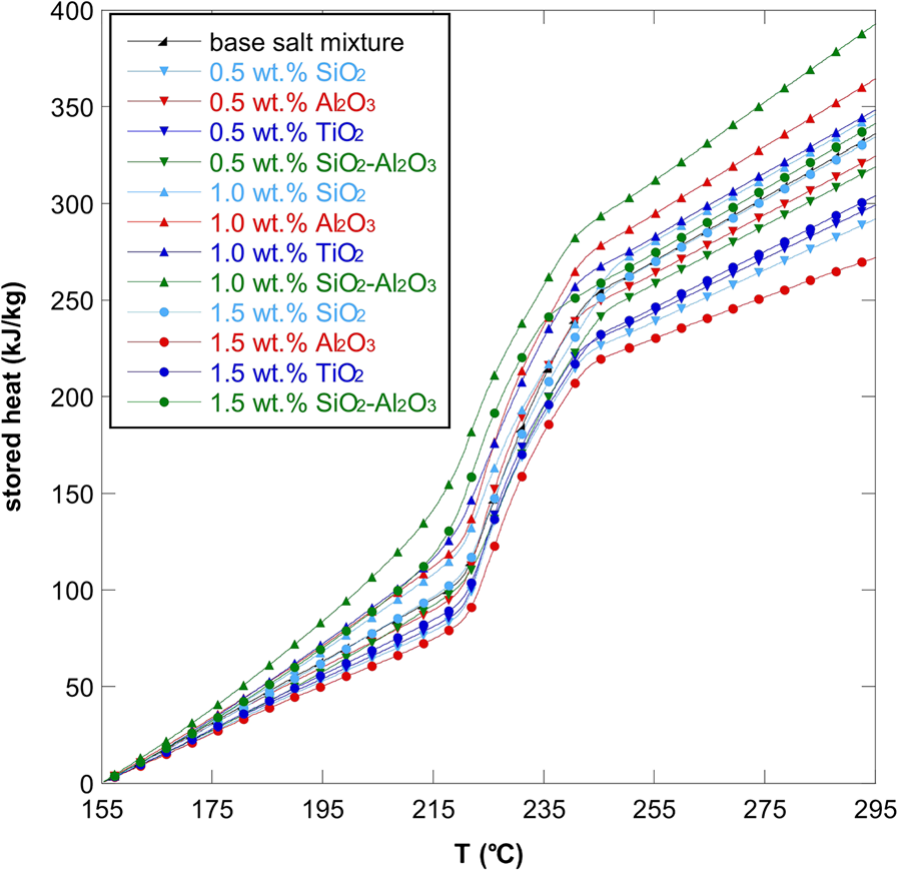
**Stored heat versus temperature for NaNO**_**3**_-**KNO**_**3 **_**binary salt mixture and nanofluids.** Obtained with base salt and 0.5, 1.0, and 1.5 wt.% of oxide nanoparticles.

The data obtained from DSC tests were also elaborated in order to evaluate the stored heat as a function of the temperature and these results are reported in Figure 5 for all the nanofluids studied and for the three concentrations considered. It is easy to understand the real effect of the nanoparticles on the storage capacity of the material. In fact, once a minimum (solid phase) and a maximum (liquid phase) working temperature was fixed, the storage capacity of the material was supplied from the integration of the curve under consideration.

The area enclosed by a curve of the nanofluid and the curve of the base salt provided an indication of how much the thermal capacity of the new storage medium was increased. The curves related to the nanofluids with 0.5 wt.% and 1.5 wt.% of nanoparticles were lower than the base salt curve. On the contrary, the curves of stored heat versus temperature for the nanofluids with 1.0 wt.% of nanoparticles were above the base salt curve. Even in this case, it was evident the higher increase of the thermal capacity obtained with the addition of 1.0 wt.% SiO_2_-Al_2_O_3_ nanoparticles. The total gain due to the introduction of the nanoparticles was given by the ratio between the total stored heat of the nanofluids and the total stored heat of the base salt mixture. The stored heat as function of temperature avoids the definition of a single melting temperature, a constant heat capacity that might not exist, as well as the separation between sensible heat and latent heat [48].

### Morphological investigation

The morphological characterization was carried on in order to evaluate the degree of dispersion of the nanoparticles in the nanofluid after the calorimeter cycle test was performed. SEM analysis was useful to understand the factors that are responsible of the improvement of the C_p_. In fact, it is assumed that a uniform distribution and a homogeneous dispersion of the filler contribute to higher C_p_ values and lower melting points of the nanofluid.

**Figure 6:**
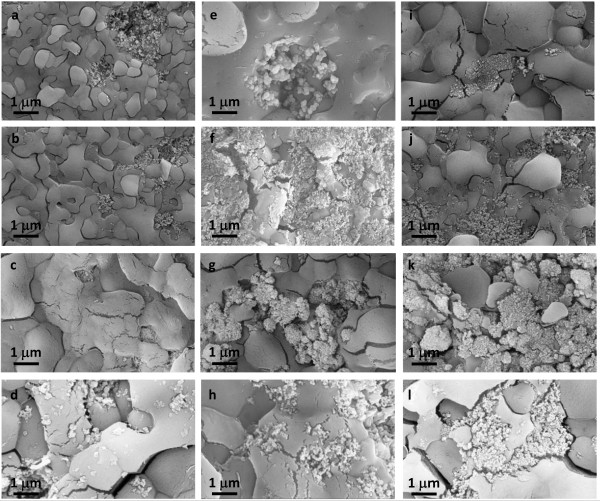
**SEM micrographs of nanofluids based on NaNO**_**3**_**-KNO**_**3 **_**with 0**.**5 wt.% (a,b,c,d), 1**.**0 wt.% (e,f,g,h)**, **and 1**.**5 wt% (i,j,k,l) of nanoparticles.** silica **(a,e,i)**, alumina **(b,f,j)**, titania **(c,g,k)** and alumina-silica nanoparticles **(d,h,l)**.

SEM micrographs of all nanofluids are shown in Figure 6 where panels a,b,c, and d refer to 0.5 wt.% of nanoparticles, e,f,g, and h to 1.0 wt.%, and i,j,k, and l to 1.5 wt.%. From this figure, it was observed that with the lower concentration of nanoparticles, they were not dispersed enough in the base salt. On the contrary, Figure 6i,j,k, and l showed that 1.5 wt.% of nanoparticles was a too-high concentration in order to disperse the nanoparticles. Observing SEM micrographs of the salt with 1.0 wt.% of silica nanoparticles (Figure 6e), it was possible to see the presence of non-homogenous aggregates of silica nanoparticles. Figure 6f shows the structure of nanofluids with 1 wt% of alumina. It was possible to observe Al_2_O_3_ aggregates well dispersed in the nanofluid. SEM images of the nanofluid obtained by adding 1.0 wt.% of TiO_2_ nanoparticles to the base salt mixture are reported in Figure 6g. Even in this case, the nanoparticles tended to form bigger aggregates. Finally, Figure 6d,h, and l shows the morphology of the nanofluid realized with the mixture of 82% of SiO_2_ and 18% of Al_2_O_3_. This is the nanofluid that showed the higher increase of heat capacity. In this case, the filler seems better distributed in the salt matrix especially with 1.0 wt.%, suggesting the presence of an interaction between the SiO_2_-Al_2_O_3_ nanoparticles and the salt that could be responsible of the specific heat enhancement.

The agglomeration observed in the nanofluids could be caused by variations in pH during the synthesis of nanofluids, variations in concentration or temperature cycling [49]. Moreover, the distribution of nanoparticles in the base fluid could be improved using surfactants to break down the particle conglomeration in suspension [50]. This is the subject of future work by our research group.

The SEM characterization of the nanofluids developed in our study did not show the presence of a substructure at nanoparticle locations as reported in other researches [21,32] that was considered as probably responsible for heat capacity enhancement. Nevertheless, the higher specific heat recorded in our research could be associated to the high specific surface energies associated with the high surface area of the nanoparticles per unit volume [51,52]. Moreover, the higher enhancement obtained with SiO_2_-Al_2_O_3_ nanoparticles could be potentially due to the large distribution of nanoparticle size from 2 to 200 nm. This also implies the presence of smaller nanoparticles with respect to the other additives. Nanoparticles with a smaller diameter could be more effectively dispersed into the salt and give better thermal characteristics [53,54]. The size of nanoparticles, in fact, defines the surface-to-volume ratio and the suspension of smaller particles has a greater area of the solid/liquid interface (for the same volume concentrations). Hence, the contribution of interfacial effects will be stronger in this kind of suspensions [55,56].

## Conclusions

High-temperature nanofluids with the addition of different kinds of nanoparticles to a binary salt (NaNO_3_-KNO_3_ with a 60:40 ratio) with PCM behavior were studied. The nanoparticles added were silica, alumina, titania, and a mixture of silica-alumina. Three weight fractions were studied (0.5, 1.0, and 1.5 wt.%). The nanofluids obtained were subjected to calorimetry and the dispersion of the nanoparticles was analyzed by SEM. The effect on nanoparticle concentration was also investigated.

DSC showed that the addition of nanoparticles to base salt increased the heat of fusion of the nanofluid compared to the observed heat of fusion of the unmodified base salt (with the higher increase obtained with 1.0 wt.%). The onset temperature and the melting temperature were lowered by the addition of all nanoparticles. Moreover, there was an increase of C_p_ with the addition of 1.0 wt.% of nanoparticles. In particular, the best results were obtained with the addition of 1.0 wt.% of SiO_2_-Al_2_O_3_ that showed a decrease of more than 8°C for the melting point and 10°C for the onset temperature and higher enhancement of the specific heat capacity of the salt mixture (57% in solid phase and 22% in liquid phase).

SEM images suggest a higher interaction between the SiO_2_-Al_2_O_3_ nanoparticles and the salt especially with 1.0 wt.% of nanoparticles. This work demonstrates that an increase in specific heat is achievable with the use of nanoparticles with relevant advantages for thermal storage. In general, concentrations lower and higher than 1.0 wt.% did not show the same enhancement of thermal properties. Moreover, the observed enhancements are suggested to be due to the high specific surface energies associated with the high surface area of the nanoparticles per unit volume.

The enhanced specific heat capacity of the nanofluids can significantly reduce the required amount of thermal energy storage media in concentrated solar power plants with a consequent reduction in the cost of electricity.

## Abbreviations

TES: Thermal energy storage; LHTES: Latent heat thermal energy storage; PCM: Phase change material; DSC: Differential scanning calorimetry; SEM: Scanning electron microscopy.

## Competing interests

The authors declare that they have no competing interests.

## Authors’ contributions

MC designed the experiments and wrote the paper. GC prepared the samples and performed the tests. MC and AM analyzed the data. JK participated in the coordination of the study and in the redaction of the manuscript. All authors read and approved the final manuscript.
